# Commonly reported negative experiences on social media are associated with poor mental health and well-being among adolescents: results from the “LifeOnSoMe”-study

**DOI:** 10.3389/fpubh.2023.1192788

**Published:** 2023-06-02

**Authors:** Jens Christoffer Skogen, Amanda Iselin Olesen Andersen, Turi Reiten Finserås, Priya Ranganath, Geir Scott Brunborg, Gunnhild Johnsen Hjetland

**Affiliations:** ^1^Department of Health Promotion, Norwegian Institute of Public Health, Bergen, Norway; ^2^Centre for Evaluation of Public Health Measures, Norwegian Institute of Public Health, Oslo, Norway; ^3^Alcohol and Drug Research Western Norway, Stavanger University Hospital, Stavanger, Norway; ^4^Department of Psychosocial Science, University of Bergen, Bergen, Norway; ^5^Center for Alcohol and Drug Research, Aarhus University, Aarhus, Denmark; ^6^Department of Alcohol, Tobacco and Drugs, Norwegian Institute of Public Health, Oslo, Norway; ^7^Department of Clinical Neuroscience, Karolinska Institutet, Stockholm, Sweden

**Keywords:** social media, adolescents, mental health, negative experiences, cybervictimization

## Abstract

**Introduction:**

Cyberbullying has been extensively studied and is associated with adverse mental health outcomes in adolescents. However, adolescents may also experience a range of other negative experiences, such as name-calling, threats, exclusion, and unwanted attention or contact from others. Few studies have investigated how adolescents’ mental health is affected by these relatively common and less severe types of negative experiences on social media (SOME). To assess the association between mental health outcomes and two aspects of negative experiences on SOME; unwanted attention and negative acts and exclusion.

**Methods:**

This study is based on a survey conducted in 2020/21 consisting of 3,253 Norwegian adolescents (56% female, M_age_ = 17 years). Eight statements about negative experiences on SOME were asked and combined into two composite measures: “Unwanted attention from others” and “Negative acts and exclusion.” Dependent variables in regression models were symptoms of anxiety, symptoms of depression, and mental well-being. Covariates in all models included age, gender, subjective socioeconomic status, and amount of SOME-use.

**Results:**

Both “negative acts and exclusion” and “unwanted attention from others” on SOME were consistently positively associated with self-reported symptoms of depression and anxiety, and negatively associated with mental well-being in both crude and adjusted analysis.

**Discussion:**

The results are indicative of an important relationship between experiencing negative events on SOME, even presumably less severe events, and worse mental health and well-being. Future research should extricate the potential causal relationship between negative experiences on SOME and mental health, as well as exploring potential precipitating and intermediating factors.

## 1. Introduction

Social media is widely used and constitutes an important aspect of everyday life, particularly for adolescents (1). The extensive use of social media among adolescents has caused concern about potentially negative effects on adolescents’ mental health and well-being (2). Although early research investigated the effect of overall amount of social media use on adolescents’ well-being, the current consensus is that social media use entails a wide range of activities and experiences that must be considered separately (3, 4).

One concern with social media platforms is that they might extend the areas where one can have negative experiences. The pervasiveness of smartphone ownership and social media use has transformed when and where relational aggression may occur (5), and cyberbullying has been extensively studied (6, 7). Bullying is a specific form of peer aggression that is repeated, intentional, and with the purpose of harming the victim, and where there is a power imbalance in favor of the perpetrator (8). Although social media can create new opportunities for bullying (2), traditional bullying seems to be more prevalent than cyberbullying (9). The association between bullying and negative mental health outcomes is well-established [e.g. (10–12),], and cyberbullying is also associated with negative mental health outcomes (5, 13–15). In fact, a meta-analysis showed that cyberbullying is more strongly related to suicidal ideation than traditional bullying (16). This might be because cyberbullying can include anonymity, a larger audience, and difficulty keeping distance from the bullying (17). In addition to experiences classified as bullying (i.e., meet the Olweus criteria), adolescents can have a range of other negative experiences. These include name-calling, threats, and exclusion, and are often termed cybervictimization. This term is less stringent than cyberbullying, and has been defined as “the broad range of intentional acts of aggression and victimization that occur through social media” [(18), p. 298].

Virtual communities that enable adolescents to build a sense of belonging can also exclude and ostracize many others. The phenomenon of cyberostracism (19, 20), where individuals are ignored or excluded during online interactions, leads to adolescents feeling angry and aggressive (21), and even victimized leading to feelings of unworthiness and depression (22). In a recent study of cyberostracism and well-being in a Chinese university context (23), cyberostracism undermined their well-being and their need satisfaction, which is the essential source for facilitating a fully functioning state (24). These deleterious effects of cyberostracism pose a threat both to the adolescent and to society by increasing negative emotional, behavioral, and anti-social responses (25).

In a study including almost 25,000 adolescents in Spain and Ecuador, Rodríguez-Hidalgo et al. (26) explored the prevalence of cybervictimization, and found that 9% experienced one form of cybervictimization at least once or twice a month. A study of lifetime prevalence among adolescents found that 59% had experienced at least one type of relational aggression on social media, such as the spreading of false rumors or name calling (27). In a Norwegian survey of adolescents, 31% reported that others had written nasty comments to them online, during gaming, or on social media in the past year (28). Furthermore, 26% reported that someone had been mean or bullied them, 24% that they had been excluded or prevented from participating in an online group, while 15% had been threatened, and 14% that someone had posted a photo of them that made them feel sad or angry. There were gender differences in these negative online experiences. Bullying, nasty comments, and threats were more common among 15–18 year old males than females (28). Conversely, more females than males had experienced being excluded from online groups or that someone had posted a photo of them that made them sad or angry.

Other negative interactions on social media can include unwanted attention or contact from others, as one becomes approachable to a wide range of people. A Norwegian report showed that three out of 10 13–18 year-olds have received sexual comments in the past year, increasing from 19% among 13–14 year-olds to 42% among 17–18 year-olds (28). While some found such comments exciting, over one third of females found it uncomfortable and half had blocked the person who had made the comment.

While cyberbullying has received extensive attention in research (2, 6, 7), there has been little focus on subjective negative experiences that less readily classify as bullying. Our own study found that low socioeconomic status was associated with more negative experiences on social media in a sample of adolescents (29). Another study using the same dataset found that both the amount of negative experiences and the number of different negative experiences was associated with increased alcohol use and indications of potential alcohol-related problems (30). A study by Rosenthal and colleagues found an association between different negative experiences on Facebook and symptoms of depression, which was consistent after controlling for socioeconomic status (31). Due to the pervasive use of social media among adolescents, investigating the relationship between these types of negative experiences on social media and mental health and well-being is warranted. In the present study, we assessed the association between mental health outcomes and two aspects of negative experiences on social media, namely unwanted attention and negative acts and exclusion. Specifically, we hypothesized positive associations between negative experiences on social media and symptoms of anxiety and depression, and a negative association with mental well-being. Based on previous research, we included age, gender, subjective socioeconomic status, and amount of social media use as covariates.

## 2. Methods

### 2.1. Sample

The present study employed cross-sectional data from the “LifeOnSoMe”-study, which recruited all public high school students in the municipality of Bergen (Norway) aged at least 16 years. One of the objectives of the “LifeOnSoMe”-study was to collect questionnaire data to shed light on adolescents’ use of social media beyond mere amount of use. Briefly, the study included several aspects of social media use, including motivations underlying use, positive and negative experiences related to use, views and opinions about their own and others use of social media, as well as how and when they use social media. Another objective of the study was to investigate the potential relationship between different aspects of social media use and other important areas in adolescent’s lives, including but not limited to mental health and well-being.

The data was collected in September and October 2020 and in June to September 2021, with a participation rate of 53 and 35.4%, respectively. The data collection was web-based, and the high school students received a survey-specific web address containing written online information about the study as well as the possibility to consent to participate. The study was approved by the regional ethics committee and was in agreement with the General Data Protection Regulation (See also “Ethics” below for more information). The total number of participants eligible for the present study was *N* = 3,253.

### 2.2. Exposure: negative experiences on social media

Eight statements regarding negative experiences on social media were asked, each with responses ranging from “Never” to “Very often.” The statements did not have a specified timeframe. The statements are derived from analyses of focus group interviews of adolescents regarding social media use and mental health and well-being (32). In brief, the focus group interviews investigated the perspectives and insights of adolescents regarding the positive and negative associations between social media use and their mental health and well-being. The participants were recruited from two senior high schools, and three main themes are presented in Hjetland et al.’ (32) paper: “Interpersonal consequences of social media,” “personal consequences of social media,” and “motivations affecting social media use,” each with a set of sub-themes. Afterward, the qualitative data were used to develop a set of statements reflecting the content and meaning of the described themes to be used in questionnaires. The statements related to negative experiences on social media and the corresponding response distributions are presented in Table 1.

**Table 1:** Negative experiences on social media.

Statements	*N* = 3,253
#1: I get unwanted attention from strangers	
Never	33.7%
Seldom	33.4%
Sometimes	23.6%
Often	6.7%
Very often	2.7%
#2: Others share pictures/videos of me against my will	
Never	59.7%
Seldom	30.5%
Sometimes	8.1%
Often	0.9%
Very often	0.8%
#3: I receive unwanted nude pictures/sexualised content	
Never	53.8%
Seldom	22.5%
Sometimes	17.0%
Often	4.3%
Very often	2.4%
#4: I am asked to send nude pictures/sexualised content	
Never	60.3%
Seldom	20.0%
Sometimes	13.3%
Often	4.2%
Very often	2.2%
#5: I get negative comments on what I post	
Never	84.5%
Seldom	10.8%
Sometimes	3.8%
Often	0.5%
Very often	0.3%
#6: I receive unpleasant or hurtful messages	
Never	74.7%
Seldom	16.8%
Sometimes	7.0%
Often	0.7%
Very often	0.7%
#7: Others say/post negative things about me	
Never	74.7%
Seldom	16.6%
Sometimes	7.0%
Often	1.1%
Very often	0.6%
#8: I feel excluded from groups/chats	
Never	62.0%
Seldom	24.5%
Sometimes	10.0%
Often	2.1%
Very often	1.4%

Statements and response distribution (%).

Based on previously published findings from the same dataset (29), the variables 1, 3, and 4 were combined as a composite measure of “Unwanted attention from others” (Cronbach’s alpha; 0.81), and the remaining five variables were combined as a composite measure of “Negative acts and exclusion” (Cronbach’s alpha; 0.84). Lastly, we also estimated the number of endorsed items (i.e., more than “never”) ranging from 0 to 8. This count variable was named “Number of negative experiences.”

### 2.3. Outcomes: mental health and well-being

#### 2.3.1. Symptoms of anxiety

Anxiety was measured by the questionnaire General Anxiety Disorder 7 [GAD-7; (33)]. GAD-7 consists of seven questions regarding symptoms of general anxiety scored from 1 (not at all) to 4 (almost every day). The questionnaire can be used as a continuous measure [total score, ranging from 0 to 21 in this study (28 maximum)] or as a dichotomous variable with a cut-off of 10 to define case-level. Cronbach’s alpha was 0.89 in the present sample. In the present study GAD-7 was used both as a continuous and a dichotomous variable (see section 3).

#### 2.3.2. Symptoms of depression

Depression was measured by the Short Mood and Feelings Questionnaire [SMFQ; (34)]. SMFQ consists of 13 statements related to symptoms of depression with the following response options 0 (not true), 1 (sometimes true), and 2 (correct). The questionnaire can be used as a continuous measure (total score, ranging from 0 to 26) or as a dichotomous variable with a cut-off at the 90th percentile to define case-level. Cronbach’s alpha was 0.91 in the present sample. In the present study, SMFQ was used both as a continuous and a dichotomous variable (see section 3).

#### 2.3.3. Mental well-being

Mental well-being was measured using the Warwick–Edinburgh Well-being Scale (35). WEMWBS consists of 14 statements related to mental well-being and quality of life scored from 1 (not at all) to 5 (all the time). WEMWBS can be used as a continuous measure (total score, ranging from 5 to 70), and as a dichotomous measure with the median as the cut-off point as recommended by those who developed the scale and empirical findings. Cronbach’s alpha was 0.92 in the present sample. In the present study, WEMWBS was used both as a continuous and a dichotomous variable (see section 3).

### 2.4. Covariates

#### 2.4.1. Age and gender

Age and gender were registered by self-report. Gender included a non-binary option, but only 37 participants ticked this option. Due to the small number, they were excluded from further analyses in the present study.

#### 2.4.2. Subjective socioeconomic status

The participants could indicate their subjective socioeconomic status (S-SES) by responding to the question “How well off do you consider your own family to be compared to others?” Response options ranged from 0 (“Very poor”) to 10 (“Very well off”). The distribution of the S-SES variable was right-skewed with mean of 7.2 (standard deviation 1.8). In the present study, a tripartite variable was created differentiating between low SES (scores 0–4), medium SES (scores 5–7), and high SES (scores 8–10).

#### 2.4.3. Amount of social media use

Two questions regarding amount of social media use were included:

1. “How often do you use social media?” with response options ranging from “Almost never” to “Almost constantly.” For the purposes of the present study, we differentiated between “Daily or less,” “Many times a day,” and “Almost constantly.”
2. “The days you use social media, approximately how much time do you spend per day?” with response options ranging from “Less than 30 min” to “More than 5 h.” In the present study, we differentiated between “Less than 2 h,” “2–4 h,” “> 4–5 h,” and “More than 5 h.”

## 3. Statistical analysis

**Figure 1:**
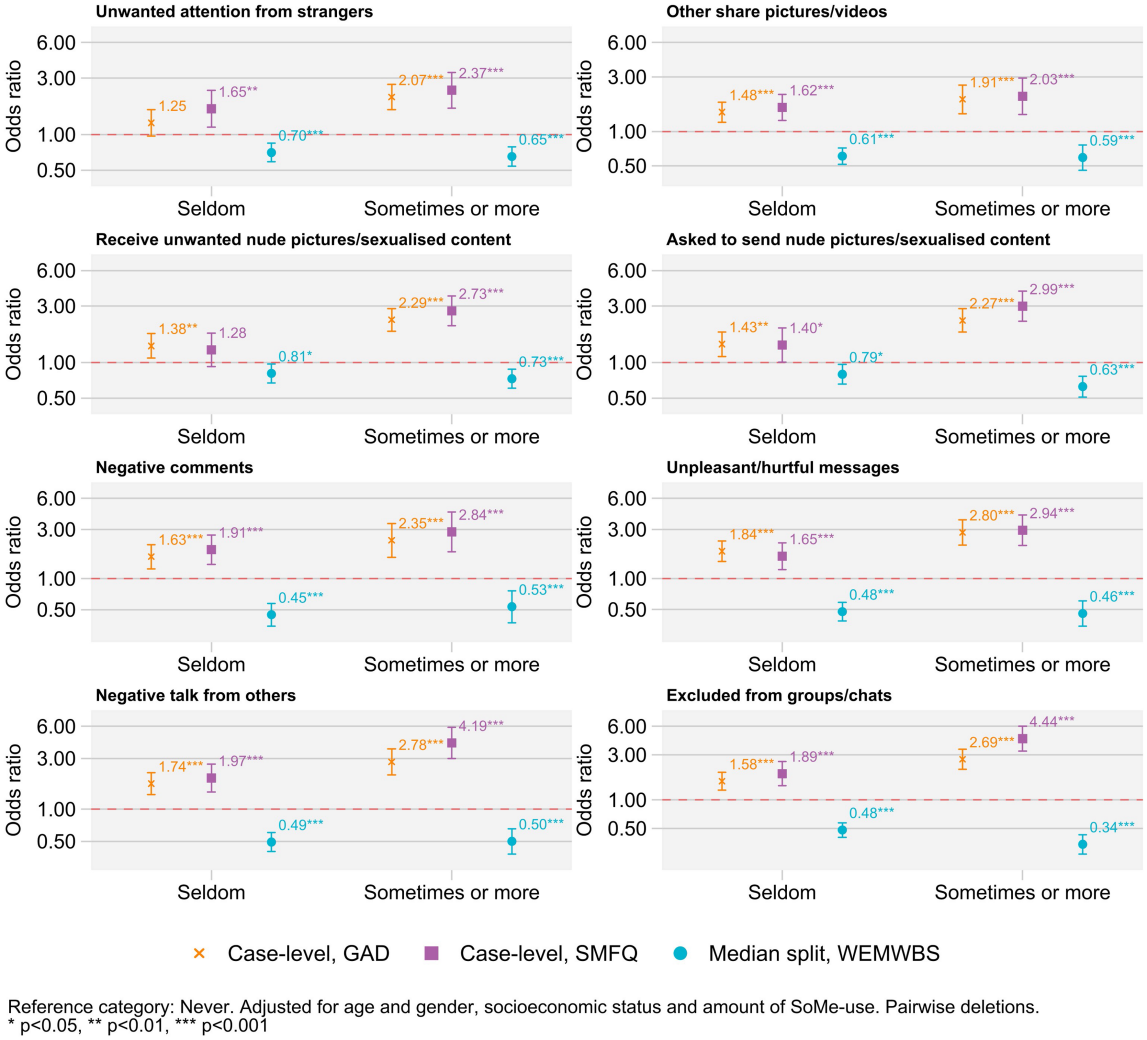
Association between mental health and well-being, and negative experiences on SoMe. Logistic regression models. Adjusted for age and gender, socioeconomic status, and amount of SoMe-use. Pairwise deletion.

First, results from descriptive analyses of the included variables are presented in Table 2, using mean and standard deviation and median and interquartile range for continuous data and frequency and proportion for categorical data. In separate logistic regression models, the three outcomes (case-level depression, case-level anxiety, and the median split of mental well-being) were sequentially regressed on the variables “negative acts and exclusion,” “unwanted attention from others,” and “number of negative experiences.” The results are presented as odds ratios with 95% confidence intervals in Table 3. In addition to the results from the logistic regression models, we also present the *E*-values for each model in Table 3. The E-value gives an estimate of the amount of unmeasured confounding needed to be present in order to fully explain away the observed associations, conditional on the measured covariates (16). As such, the *E*-values gives a numerical value to the liminal additional confounding necessary to reach non-significance. The associations between negative experiences on social media and symptoms of depression, symptoms of anxiety and mental well-being score were estimated using linear regression models across the variables “negative acts and exclusion” and “unwanted attention from others” and the results are presented in Table 4. The three outcomes were converted to *z*-scores, to obtain *y*-standardized regression coefficients. Finally, the association between each of the separate variables related to negative experiences and case-level depression, case-level anxiety, and mental well-being (median split) was estimated using logistic regression models and presented in Figure 1. All regression models included age, gender, subjective socioeconomic status, and amount of social media use as covariates. Data handling and analyses were performed in Stata version 15, and statistical analyses was performed both in Stata version 15 (36) and in R (37). Tables were produced using the “gtsummary” package in R (38). *E*-values were calculated using the *E*-value command available for Stata (39).

**Table 2:** Summary statistics of included variables.

Characteristic	*N* = 3,253^1^
Age	
16	593 (18%)
17	1,556 (48%)
18	894 (27%)
19+	210 (6.5%)
Gender	
Boys	1,418 (44%)
Girls	1,835 (56%)
Subjective socioeconomic status	
Low (0–4)	209 (6.4%)
Medium (5–7)	1,704 (52%)
High (8–10)	1,340 (41%)
How often do you use SoMe?	
Every day or less	771 (24%)
Many times a day	1,630 (50%)
Almost constantly	852 (26%)
Time spent on SoMe?	
Less than 2 h	969 (30%)
2–4 h	1,228 (38%)
>4–5 h	587 (18%)
More than 5 h	469 (14%)
Number of negative experiences on SoMe	3.00 (1.00, 5.00)
Negative acts and exclusion, SoMe	1.41 (0.57)
Unwanted attention from others, SoMe	1.86 (0.88)
Score on WEMWBS	48 (10)
Score on GAD	5.0 (2.0, 8.0)
Score on SMFQ	5 (3, 10)

1 *n* (%); Median (IQR); Mean (SD).

**Table 3:** Association between negative experiences and case-level depression, case-level anxiety, and well-being (median split).

	Case-level depression				Case-level anxiety				Well-being, median split			
Characteristic	OR^1^	95% CI^1^	*p* value	*E*-value	OR^1^	95% CI^1^	*p* value	*E*-value	OR^1^	95% CI^1^	*p* value	*E*-value
Negative acts and exclusion, SoMe	2.29	1.93, 2.72	<0.001	4.01	1.91	1.65, 2.20	<0.001	2.11	0.49	0.42, 0.57	<0.001	2.21
Unwanted attention from others, SoMe	1.74	1.54, 1.98	<0.001	2.88	1.56	1.41, 1.73	<0.001	1.81	0.80	0.73, 0.88	<0.001	1.48
Number of negative experiences on SoMe	1.25	1.19, 1.31	<0.001	1.81	1.19	1.15, 1.24	<0.001	1.41	0.85	0.83, 0.88	<0.001	1.39

1 OR, odds ratio; CI, confidence interval.

Logistic regression models, adjusted for age and gender, socioeconomic status, and amount of SoMe-use.

**Table 4:** Association between negative experiences and symptoms of depresssion, symptoms of anxiety, and well-being score.

	Depression symptoms			Anxiety symptoms			Well-being, score		
Characteristic	Beta	95% CI^1^	*p* value	Beta	95% CI^1^	*p* value	Beta	95% CI^1^	*p* value
Negative acts and exclusion, SoMe	0.46	0.40, 0.51	<0.001	0.36	0.31, 0.42	<0.001	−0.37	−0.43, −0.31	<0.001
Unwanted attention from others, SoMe	0.25	0.21, 0.29	<0.001	0.24	0.20, 0.28	<0.001	−0.13	−0.17, −0.09	<0.001

1 CI, confidence interval.

Dependent variables Z-scored, beta equivalent to standard deviation. Linear regression models, adjusted for age and gender, socioeconomic status, and amount of SoMe-use.

## 4. Results

Summary statistics of the included variables are presented in Table 2. The median age was 17 years, and 56% of the sample were female. Just over half of the sample reported medium socioeconomic status. Half of the sample reported using social media many times a day, and 70% reported using social media at least 2 h a day. Almost three out of four (74.5%) reported at least one of the negative experiences seldomly or more frequently, and the median number of different negative experiences was 3.

Reporting at least one of the negative experiences at least seldomly was associated with increased odds for case-level anxiety [Odds ratio (OR) 2.16 (95% CI: 1.74–2.69), *p* < 0.001] and case-level depression [OR 2.26 (1.67–3.04), *p* < 0.001], and lower odds for above median mental well-being [OR 0.57 (0.49–0.66), *p* < 0.001].

All of the three main measures of negative experiences on social media were positively associated with case-level depression (ORs ranging from 1.25 to 2.29) and case-level anxiety (ORs ranging from 1.19 to 1.91). For mental well-being, negative associations were observed (ORs ranging from 0.85 to 0.49). The *E*-values ranged from 1.39 to 4.01. The highest *E*-value was observed for the association between “negative acts and exclusion” and case-level depression, while the lowest *E*-value was observed for number of negative experiences and the median-split of mental well-being. See Table 3 for further details.

Both “negative acts and exclusion” and “unwanted attention from others” on social media were positively associated with symptoms of depression [0.46 standard deviations (SD) and 0.25 SD, respectively] and anxiety [0.36 and 0.24 SD, respectively], and negatively associated with score on mental well-being (−0.37 and −13SD, respectively). The strength of the observed association (i.e., “effect size”) ranged between small to medium. See Table 4 for further details.

Results from separate logistic regression models for all the specific negative experiences on social media are presented in Figure 1. Across all the variables gauging negative experiences on social media, positive association with case-level depression and anxiety were observed, while a negative association was observed with mental well-being (median split). All estimates were statistically significantly different from the reference category “never,” except for case-level depression and reporting “receive unwanted nude pictures/sexualized content” seldom, and case-level anxiety and reporting “unwanted attention from strangers” seldom. Results from crude logistic and linear regression models yielded similar results to the presented findings (data not shown).

## 5. Discussion

In the present study, we examined the potential association between a range of common negative experiences on social media and mental health and well-being. Our results indicate consistent positive associations between both “negative acts and exclusion” and “unwanted attention from others” on social media and self-reported symptoms of depression and anxiety. There were also consistent negative associations between both types of negative experiences and mental well-being. The same pattern was seen for the number of different negative experiences on social media and mental health and well-being. All the investigated associations were robust to adjustments for age, gender, socioeconomic status, as well as amount of social media use. The estimates for what levels of unmeasured confounding were needed to explain away our observed associations varied from small to strong. For the association with the highest point estimate, between “negative acts and exclusion” and case-level depression, the observed odds ratio of 2.29 could be explained away by an unmeasured confounder that was associated with both the exposure and the outcome by an odds ratio of 4.01-fold each, above and beyond the measured confounders (40). Weaker confounding could not do so. Although the other *E*-values were lower, our overall consistent findings can be considered robust to any reasonably expected impact of unmeasured confounding. Overall, the presented results are indicative of an important relationship between experiencing negative events on social media, even presumably less severe events, and worse mental health and well-being.

Our findings are in line with a previous study investigating negative experiences on Facebook and association with depressive symptoms among young adults (31). The paper by Rosenthal and colleagues included similar experiences to what we studied, such as “meanness” and “unwanted contact.” They also found that all the included negative experiences were related to depressive symptoms. Interestingly, they found that past year negative experiences was less strongly associated with depressive symptoms compared to lifetime negative experiences, which the authors interpret as being an indication that experiencing these kinds of events in adolescence is more distressing than in adulthood. Similarly, our findings also support the finding from Niu and colleagues that there is a positive correlation between cyber-ostracism and symptoms of depression among adolescents aged 12–18 years (22). Taken together, the evidence available indicates that the day-to-day interactions and events on social media have a real-life impact on its users (22).

### 5.1. Strengths and limitations

The present study holds several strengths. First, the items related to negative experiences on social media cover many different aspects and are derived from focus group interviews with adolescents about their motivations, perspectives, and experiences related to the use of social media (32). It is therefore reasonable to believe that this ensures relevance and a high ecological validity. Second, the outcomes included are widely used and validated age-appropriate measures of potential mental health problems and mental well-being. Third, the robust sample size allowed for inclusion of potentially important confounders, and we also estimated the amount of unmeasured confounding needed to render the associations non-significant using the E-value approach (40). Fourth, the study used a broad definition of social media, and was not restricted to one or a few popular platforms.

Several limitations also need to be noted. First, the study is cross-sectional, which limits our ability to draw inferences about directionally. Second, although a range of different negative experiences on social media are covered, the list of experiences is not exhaustive, and it is quite possible that other commonly occurring experiences are also associated with mental health outcomes. Third, although we asked about how often they have lived through the listed experiences (ranging from “never” to “very often”), we did not provide any timeframe for them to consider. As such, the answers given could in theory be life-time experiences. However, due to recall bias, it is more likely that they would remember more recent experiences or experiences which had a greater impact on them (e.g., particularly more emotionally upsetting events). Relatedly, we did not include any measure of how the experiences impacted them. Fourth, we only included symptoms of anxiety and depression, as well as mental well-being as outcomes in our study. Thus, the potential relation between negative experiences on social media and other important aspects of mental health is not covered in the present study. Fifth, the participation rate was less than optimal, potentially decreasing the generalizability of our findings. The participation rate may also bias the reported descriptive statistics but is more unlikely to have substantially biased the reported associations (41). Lastly, the adolescents included are aged 16+, and the findings reported are not necessarily valid for other age groups such as younger adolescents or young adults.

### 5.2. Implications and future research

The findings from the present study indicate that negative experiences on social media are relatively common, as almost three out of four reported at least one of the included negative experiences seldomly or more frequently. Our findings also consistently indicate a relationship between these negative experiences and mental health problems and mental well-being. The combination of a relatively high prevalence of these experiences and the consistent associations found is noteworthy. Also, our findings are robust considering our estimate of the impact of unmeasured confounding. That is, it is unlikely that all of our reported associations would be rendered non-significant by introducing additional co-variates. Given our results, it is reasonable to surmise that the impact of negative experiences on social media on mental health is of public health relevance and should be addressed accordingly. Increased attention should be given not only to severe and uncommon negative experiences on social media, but also to more commonly reported negative experiences such as social exclusion and negative feedback/backbiting. A previous study has for instance shown that common life events/difficulties such as interpersonal difficulties in general are associated with mental health problems (42). The authors of that paper recommended—as do we—that adolescents should be helped by increasing their coping skills and resilience to manage various kinds of adversities they are likely to face. This notion is further supported by findings that resilience and social support potentially play a pertinent mediating role in the observed relationship between negative life events and quality of life among adolescents (43). Furthermore, specifically in relation to negative experiences on social media, there is a need to increase social media literacy among adolescents and parents as well as increasing engagement from parents and teachers regarding the lives adolescents live on social media. The latter recommendation may facilitate communication and increase awareness regarding how social media experiences, both positive and negative, affect the day-to-day mood and behavior of adolescents. If not, the negative repercussions of even presumably less severe but commonly experienced negative events could be longstanding and persist into later stages of development.

Finding ways to strengthening the potentially positive aspects of social media use, such as socializing and interacting with peers should also be emphasized in future research and in the development of intervention programs related to mental health among adolescents. Our own research has for instance found that social media can serve as an arena for social support among adolescents, and that social support instigated in digital settings is positively related to mental health and well-being (44). As such, health-promoting interventions aimed at increasing both social media literacy and prosocial interaction on social media may be beneficial also in relation to negative experiences on social media. It is our view that it is unlikely to fully safeguard oneself from negative experiences on social media. We therefore believe that interventions specifically targeting negative experiences in tandem with more general health-promoting principles would hold the most promise.

Regarding future research, several aspects related to our findings need to be addressed. As with all cross-sectional studies, we are not able to ascertain potential causality between negative experiences on social media and mental health. Future research should employ longitudinal designs to investigate the putative causal relationship between the exposures and mental health. While it is likely that the negative experiences examined in the present study influence mental health adversely, it is also possible that poor mental health elicits social media use and interactions with others on social media that increases the likelihood of negative experiences. By extension, this can serve as the start of a vicious cycle where both negative experiences and certain mental health problems fuel each other to the detriment of the individuals in question. Future research should also aim to investigate potential intermediate factors potentially mitigating the relationships reported here. There is also a need to better understand the plethora of features and functionality of different social media platforms to shed light on how they may facilitate, and even encourage, negative and harmful behavior and social interactions. Lastly, there is also a need to investigate the role of individual differences and developmental trajectories to shed light on the dynamics of the associations in a longer-term perspective.

## 6. Conclusion

In the present study, we found consistent associations between a range of commonly reported negative experiences on social media and symptoms of anxiety and depression as well as reduced mental well-being. Taken together with previous findings, our findings point to a potentially important public health issue, which needs to be addressed. Health-promoting intervention programs aimed at strengthening social media literacy and prosocial interaction on social media may be one way to specifically prevent negative experiences. In addition, a strengthening of the adolescents’ resilience in the face of adversity would probably also be beneficial. Future research should try to extricate the potential causal relationship between negative experiences on social media and mental health, as well as exploring potential precipitating and intermediate factors.

## Data availability statement

The data analyzed in this study is subject to the following licenses/restrictions: explicit consent from the participants is required by the Norwegian Health research legislation and the Norwegian Ethics committees in order to transfer health research data outside of Norway. Ethics approval for this study was also dependent on storing the research data on secure storage facilities located at the Norwegian Institute of Public Health, which prevents the authors from providing the data as supplementary information. Requests to access these datasets should be directed to jens.christoffer.skogen@fhi.no.

## Ethics statement

This study involving human participants were reviewed and approved by Regional Ethics Committee (REK) in Norway (REK#65611). Written informed consent from the participants' legal guardian/next of kin was not required to participate in this study in accordance with the national legislation and the institutional requirements.

## Author contributions

JS: conceptualization, formal analysis, and writing and editing all drafts. AA and TF: review and editing, feedback on analytical approach, and literature review. PR: review and editing and literature review. GB: review and editing and feedback on analytical approach. GH: conceptualization, review and editing, feedback on analytical approach, and literature review. All authors contributed to the article and approved the submitted version.

## Funding

The work of GH was supported by Dam Foundation (grant number 2021/FO347287) while the work of JS, AA, and TF was supported by The Research Council of Norway (grant number 319845). PR and GB received no external funding for the research, authorship, and/or publication of this article.

## Conflict of interest

The authors declare that the research was conducted in the absence of any commercial or financial relationships that could be construed as a potential conflict of interest.

## Publisher’s note

All claims expressed in this article are solely those of the authors and do not necessarily represent those of their affiliated organizations, or those of the publisher, the editors and the reviewers. Any product that may be evaluated in this article, or claim that may be made by its manufacturer, is not guaranteed or endorsed by the publisher.
